# The importance of scientific competencies in German medical curricula - the student perspective

**DOI:** 10.1186/s12909-018-1257-4

**Published:** 2018-06-19

**Authors:** Antonius Ratte, Simon Drees, Tabea Schmidt-Ott

**Affiliations:** 1 German Medical Students’ Association (bvmd e.V.), Robert-Koch-Platz 7, 10115 Berlin, Germany; 2 University of Heidelberg, Heidelberg, Germany; 3 Charité - Universitätsmedizin Berlin, Berlin, Germany; 4 University of Oldenburg, Oldenburg, Germany; 5 London School of Economics and Political Science (LSE), London, UK

**Keywords:** German medical training, Student research, Scientific competencies, Scientific training, Medical curriculum, Research skills

## Abstract

**Background:**

Scientific competencies are of great importance for physicians; not only for conducting reliable research, but also for patient care. However, there is growing concern that a lack of scientific competencies among physicians may lead to a deterioration in the quality on biomedical research. This study aims at assessing medical students’ perspectives on the implementation of scientific competency training in German medical curricula.

**Methods:**

An online survey was conducted in order to collect German medical students’ opinions on the importance of acquiring scientific competencies during their medical studies and to provide us with an assessment of their current levels of basic scientific competencies by having them conduct a self-evaluation. Moreover, we wanted to understand their perceptions of current curricular content and to receive suggestions for improving scientific competency training. Participants were reached via the mailing lists of the German Medical Students’ Association, as well as of local medical student committees, and the German Medical Students’ Associations social media channel on Facebook.

**Results:**

In total, 2380 medical students from across all 37 German medical faculties participated in the survey. The majority of students agreed that the ability to critically evaluate the relevant literature is an important competency for physicians, and that every student should conduct a research project during their medical studies. However, the students evaluated their scientific competencies as unsatisfactory, especially with regard to statistics and scientific writing. They were strongly in favor of receiving extended research training.

**Conclusion:**

Our study provides insight into German medical students’ self-perception in relation to both patient care and biomedical research, and makes recommendations for potential improvements in scientific training. The study demonstrates that scientific competencies are of great importance to medical students in Germany. Students are not lacking motivation for scientific practice and have numerous ideas for enhancing scientific teaching opportunities. Scientific training should follow a holistic approach based on three pillars: (i) a scientific core curriculum, (ii) intracurricular research projects, and (iii) special research programs for students strongly interested in medical research.

**Electronic supplementary material:**

The online version of this article (10.1186/s12909-018-1257-4) contains supplementary material, which is available to authorized users.

## Background

Scientific competencies are essential for all future physicians [1–4]. Appropriate training during their medical studies should provide students with the knowledge and skills necessary for collecting and interpreting research results, as well as carrying out research on their own. Furthermore, it might motivate students to take part in scientific research during clinical practice, enroll in PhD-programs, or even consider a career as a (clinician) scientist.

Several international publications suggest that medical students are very critical of their own abilities regarding almost all aspects of science [5, 6]. The participation of medical students in scientific research is hampered by a lack of motivation mostly resulting from inadequate access to scientific training programs and supervision [7–9]. Despite the recent adoption of a national competency-based catalogue of learning objectives that includes a dedicated chapter on scientific competencies approved by all German medical schools, no uniform standard of scientific competency training exists in Germany [10]. Medical degree programs in Germany consist of five years of basic science, clinical courses, and electives followed by a “practical year” in which students work in different healthcare settings full-time. While some medical schools do include research projects in their curricula, a thesis or research project is not legally required to graduate or practice as a physician. There is no formal title such as MD awarded upon passing the third and final state examination, as practiced in the USA, for example. Instead, graduates have to submit a doctoral thesis to be awarded the title *Dr. med*. Confusion surrounding the use of the different titles also has been discussed in the literature [11].

The majority of medical students in Germany (59.9%) prepare such a doctoral thesis in order to attain the reputable title of *Dr. med.* [12]. This thesis is mostly written alongside medical studies, and has become subject to rigorous debates questioning its methodological and scientific quality [13]. PhD programs combining medical studies and research are not common in Germany and the European Research Council does not recognize the German medical doctoral degree *Dr. med.* as being equivalent to the scientific PhD [14]. This is problematic when German medical researchers apply for international postdoctoral programs or research grants.

The aim of our study is to assess the student perspective on scientific competency training during medical studies. In this study, we define the term “scientific competency” as the ability to conduct research as well as critically analyze and interpret research results. Our questionnaire covered two main areas: the students’ impression of the importance of research competencies as they relate to future practice, and the students’ self-evaluation of their scientific competencies. Additionally, students could make suggestions on how scientific training should be improved.

## Methods

### Survey development

A voluntary anonymous online questionnaire was developed using soscisurvey.de [15]. Question writing and survey design were conducted by the authors as part of an iterative collaborative process. The questionnaire was piloted in order to remove any ambiguity, on twelve board members of the German Medical Students’ Association, whose answers were not included in the final data analysis. Some minor changes in wording were made subsequent to the responses from the pilot study.

**Fig. 1:**
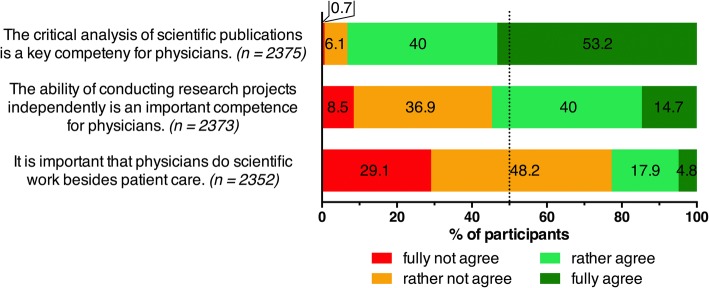
The students’ opinion on the importance of scientific competencies for physicians. Agreement on a 4 point Likert-type scale, n per item is specified in parentheses

**Fig. 2:**
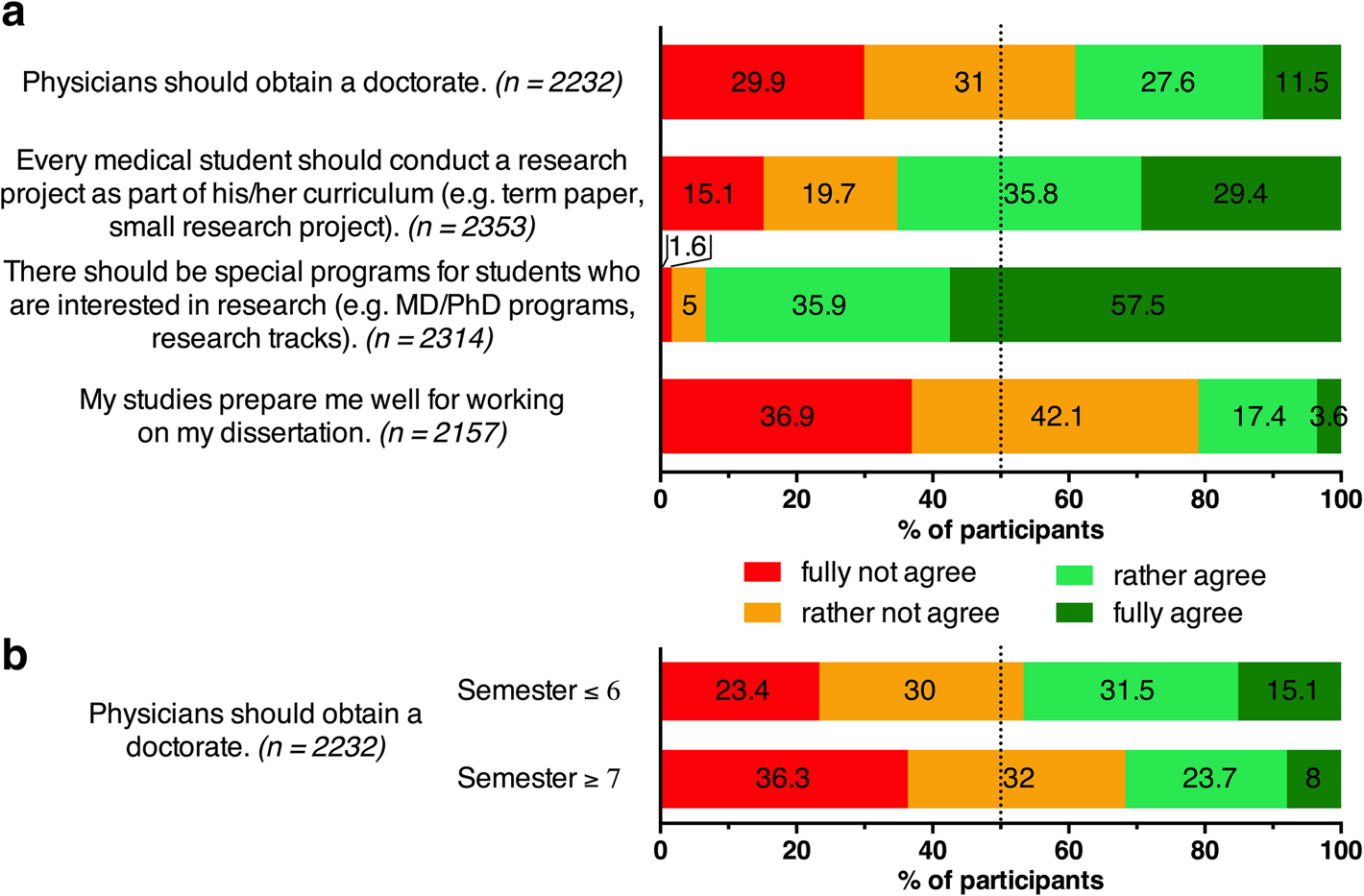
**a** The students’ opinion about the current system of medical education in Germany regarding the impartation of scientific competencies and medical doctoral degrees in Germany. Agreement on a 4 point Likert-type scale, n per item is specified in parentheses. **b** Agreement with the statement “Physicians should obtain a doctorate” of students in lower semesters (≤6 semesters) compared with students in higher semesters (≥7 semesters)

The survey took approximately ten minutes for participants to complete. It included six general questions on participants’ demographics and previous experience in research. Seven Likert-type scale questions were also included covering the following topics: (i) the importance of possessing scientific competencies as a physician and (ii) the students’ opinion about the current system of medical education in Germany in relation to the teaching of scientific competencies. Students could state their level of agreement or disagreement with different pre-formulated statements. The statements are listed in Figs. 1 and 2a. With each statement they could choose between *fully not agree*, *rather not agree*, *rather agree*, and *fully agree*.

The questionnaire also included four further Likert-type scale questions where students could evaluate their own scientific competencies on the following scale: *very bad*, *rather bad*, *rather good*, and *very good*. This self-evaluation was intended to function as an approximation of how well scientific competencies are currently imparted in medical curricula.

Another nine Likert-type questions were included to assess what changes the students felt as necessary to current curricular content for students to gain more scientific competencies. The students could state whether they demanded *much less*, *less*, *equal*, *more*, or *much more* of each content. Additionally, three open questions were included to gather students’ opinions on the following matters: (i) the best time point to start scientific research training during medical studies; (ii) what should be considered when setting up scientific education, and (iii) general suggestions for improvements. Please also refer to the questionnaire that is provided as an Additional file 1.

### Data acquisition

The participants were reached via the mailing lists of both the German Medical Students’ Association and local medical student committees. Additionally, the survey was distributed using the German Medical Students’ Association’s social media channel on Facebook. Medical students across all years of the program were invited to participate in the study. The questionnaire was available online from March to April 2015.

As answering all questionnaire items was not obligatory, only given answers were included in the analysis. The number of given answers on each item is indicated in each case.

### Data analysis

Statistical analysis was performed using Excel 2016 for Mac OS X (Microsoft, USA) and PRISM 6 (GraphPad, USA). Participants’ age and semester were summarized by calculating mean values and standard deviations. Gender, scientific experience, and the participants’ involvement in doctoral projects were summarized using percentages. The numbers of participants per medical school were analyzed. For the Likert-type scale questions, percentages were calculated. Further analysis investigated differences in the following subgroups: gender (male vs. female), semester (≤6 semesters vs. ≥7 semesters), and scientific experience (previous experience vs. no experience). To compare these subgroups, odds ratios were calculated for selected items and significance was tested using the Chi-square test. A score was derived from the answers to the four self-evaluation items to identify factors that might have influenced the students’ self-evaluation: Numeric values (0–3) were assigned to the answers of the four self-evaluation items (*very bad* = 0, *rather bad* = 1, *rather good* = 2, *very good* = 3). Adding up these values resulted in a score ranging from 0 to 12, and subgroups were compared using box-plots.

Figures were designed using PRISM 6 (GraphPad, USA). A thematic analysis of the free-text responses was conducted to analyze the students’ views on how to improve scientific education in medical school. Responses were coded and clustered based on Brown and Clarke [16].

## Results

Overall, 2380 medical students from across all 37 German medical faculties participated in the survey, covering all semesters of medical studies. The characteristics of the study population are presented in Table 1.

### Quantitative analyses

The large majority of medical students stated that it is important to critically analyze scientific work as a physician; most of them (93.2%, *n* = 2375) chose either *fully agree* or *rather agree* with the statement “The critical analysis of scientific publications is a key competency for physicians.” Nevertheless, they considered their own conduct of scientific work as less important; the statement “It is important that physicians do scientific work besides patient care” was declined by most of the participants (29.1% *fully not agree*, 48.2% *rather not agree*, *n* = 2352) (Fig. 1).

The majority of students would favor the inclusion of a small research project into their medical studies (29.4% *fully agree*, 35.8% *rather agree*, *n* = 2353). In addition, they strongly supported the establishment of specialized research programs for interested students (57.5% *fully agree*, 35.9% *rather agree*, *n* = 2314) (Fig. 2a). Only a minority of students believed that their medical studies prepared them well for working on a doctoral thesis (3.6% *fully agree*, 17.4% *rather agree*, *n* = 2157), while the majority disagreed with the statement “Physicians should obtain a doctorate” (29.9% *fully not agree*, 31% *rather not agree*, *n* = 2232). There was a tendency of students in lower semesters to express stronger support for the latter statement compared to advanced semesters (46.6% (≤6 semesters) vs. 31.7% (≥7 semesters) *fully agree* or *rather agree*; Odds Ratio = 1.88 (95% CI [1.59 to 2.24]); *p* < 0.001) (Fig. 2b).

Although the number of students pursuing a doctorate (students who stated that they had started working on their dissertation or that they were planning to start in the future) is lower among students with less support for the statement “Physicians should obtain a doctorate,” it still represents a large majority. Among students who *fully not agree* or *rather not agree* with that statement (*n* = 1360), 81% were pursuing a doctorate vs. 91.9% of students who *fully agree* or *rather agree* (*n* = 869) (Odds Radio = 0.37 (95% CI [0.28 to 0.49]; *p* < 0.001).

**Fig. 3:**
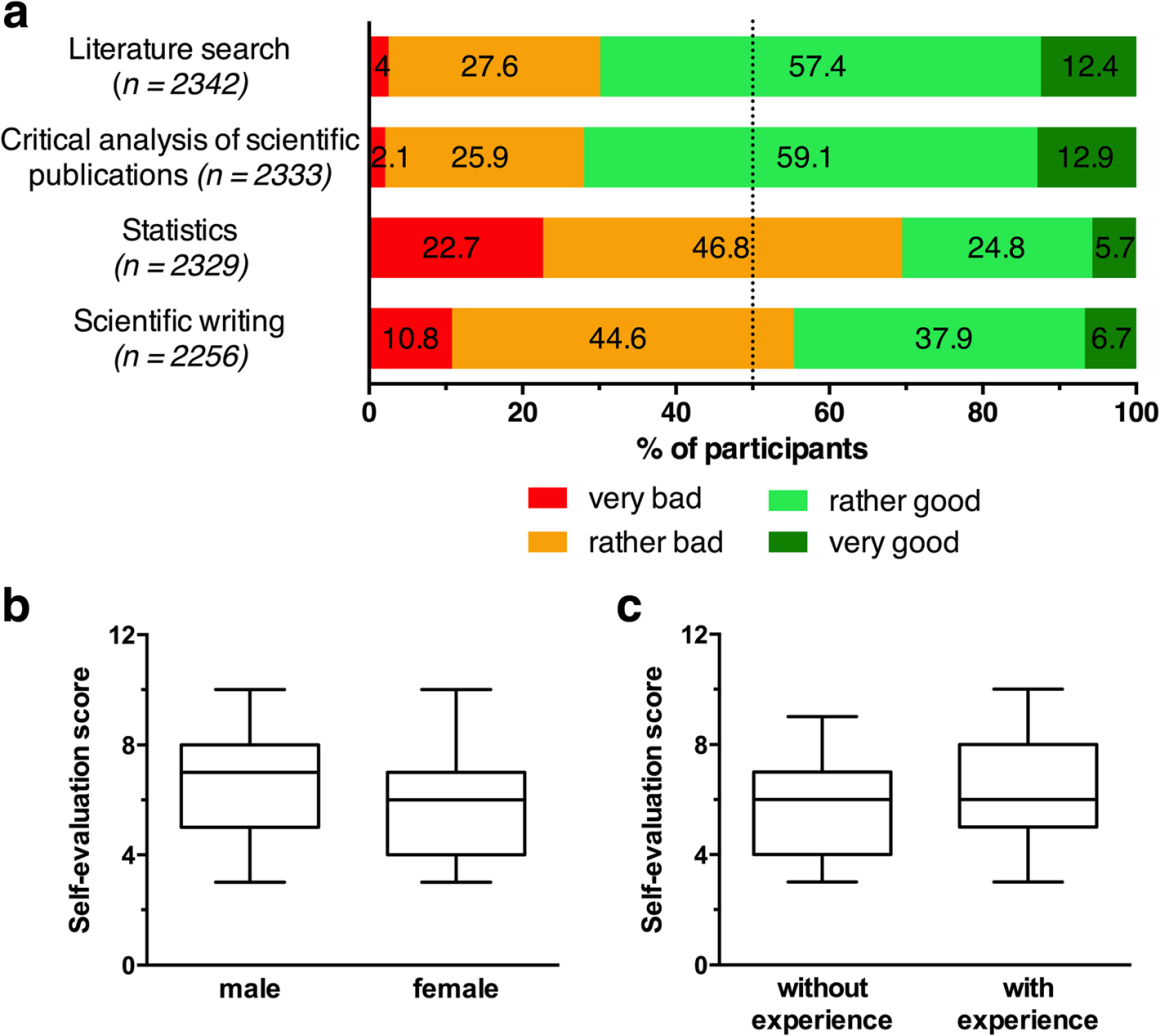
**a** The students’ self-evaluation of selected scientific competencies on a 4 point Likert-type scale, n per item is specified in parentheses. **b**, **c** Comparison of the self-evaluation of male vs. female participants and students with previous experience in research vs. students without previous experience. The self-evaluation score that is plotted on the Y axis summarizes the 4 self-evaluation items that are shown in (**a**). Boxes displays the first to third quartile, whiskers range from 5th to 95th percentile, lines within the boxes indicate the median

The students’ self-evaluation regarding selected scientific competencies was ambiguous. The majority of students rated their competencies in finding proper literature as good (12.4% *very good*, 57.4% *rather good*, *n* = 2342) and felt confident with the interpretation of scientific publications (12.9% *very good*, 59.1% *rather good*, *n* = 2333). However, the majority of students indicated insufficient competencies in statistics (22.7% *very bad*, 46.8% *rather bad*, *n* = 2329) and scientific writing (10.8% *very bad*, 44.6% *rather bad*, *n* = 2256) (Fig. 3). Overall, male participants and those with previous experience in research tended to believe that their competencies were better (Fig. 3b, c).

**Fig. 4:**
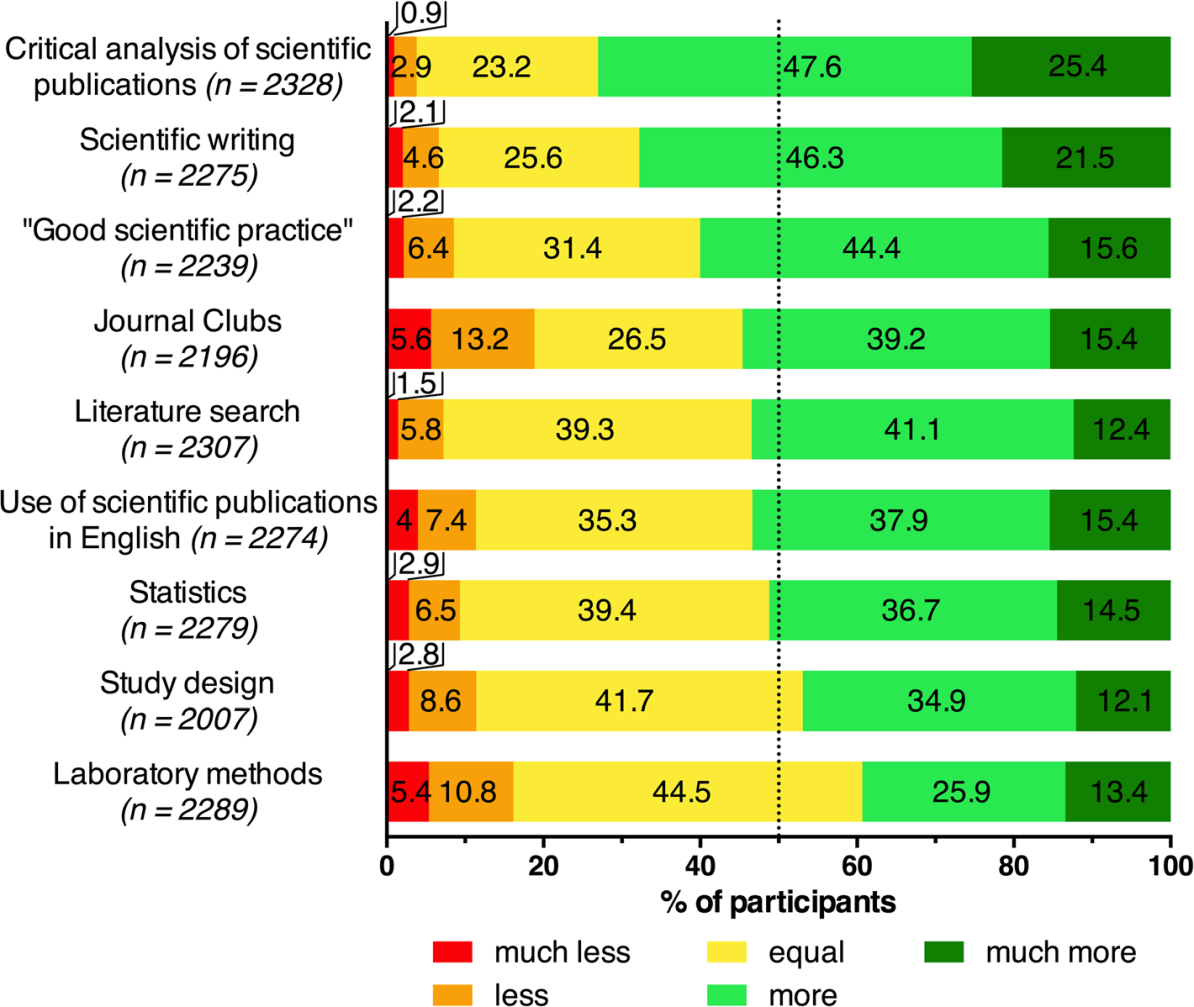
Students’ recommendations on the amount of teaching in nine proposed curricular contents. Evaluation on a 5 point Likert-type scale, n per item is specified in parentheses

Generally, the students stated that scientific training was underrepresented in medical studies and should be extended. Out of nine proposed curricular contents, seven were highly demanded by the students (Fig. 4), with the most requested content being “critical analysis of scientific publications” (73%, *n* = 2328). The majority of students recommended more “Journal Club” sessions (54.6%, *n* = 2196); however, a total of 18.8% (n = 2196) declared a wish for less “Journal Club” sessions, which made it the area with the biggest demand for reduction (Fig. 4). In contrast to the “critical analysis of scientific publications,” areas such as “study design” (47%, *n* = 2007) and “laboratory methods” (39.3%, *n* = 2289) were requested to a much lesser degree by the medical students (Fig. 4). There were no relevant differences with respect to gender, semester, or scientific experience.

### Qualitative analyses

The thematic analysis of the individual free-text responses demonstrated high ambiguity in the medical students’ opinions. On one hand, students recognized the value of intracurricular scientific research projects for improving the level of acquisition of scientific competencies. They highlighted the high relevance of scientific competencies for their future practice as physicians. One student described the situation as follows: “The importance of understanding scientific research as a physician is not communicated to the students sufficiently. It will be difficult to handle scientific data as a physician, if you haven’t conducted scientific research yourself.”

However, students considered the two pillars *research* and *clinical medicine* as entirely separate areas of expertise, explaining that it was not necessary to expand education in scientific methods. One student stated: “There is a difference between physicians (work with patients) and doctors (emphasis on research) – most of us want to be physicians and DON’T want to do research. Please, DON’T introduce more courses on research.”

*S*uggestions for improvement covered a broad variety of topics (Table 2). The students emphasized the need for improving scientific training (critical interpretation of publications should be emphasized; better supervision of scientific projects should be provided; more interactive teaching formats are required) and the general structure of medical training (students should have more freedom of choice; improvement of practical aspects of the training; limiting the medical curricula to the “essentials”). Regarding the ideal point in time to begin research training, there were no patterns observable; the suggestions ranged from the first to the last year of medical school.

**Table 1:** Characteristics of the study population (*n* = 2380)

Age (mean ± SD)	23.8 ± 3.8
Gender (%)	
Female	58.5
Male	40
n/a	1.5
Semester (mean ± SD)	6.7 ± 3.3
Number of participants per medical school	
(median and range)	45 (1–291)
Distribution of participants among medical schools	
Number of participants	Number of schools
< 5	3
5–20	10
21–100	16
> 100	8
Scientific experience (%) *(multiple answer)*	
Curriculum	12.4
Doctoral thesis	30.5
Student project	6.9
Student assistant	8.7
Other	8.7
None	49.3
Did you start working on a doctoral thesis? (%)	
Planned	40.7
Started	41.6
Cancelled	2.7
Don’t know	13
Not planned	1.9

## Discussion

Our study reveals that scientific competencies are of great importance to medical students in Germany. Students focus on basic physician competencies, such as the critical evaluation of research results. They recommend that small scientific projects should be implemented as part of their medical studies. According to the students, current medical curricula in Germany do not adequately prepare them for scientific work, and more courses on various aspects are needed to address this issue. The majority of students expressed an interest in writing a scientific thesis, but they did not believe that a doctorate degree is needed for their future work as a physician.

Various ways to tackle the lack of a next generation of medical scientists have been discussed in the national literature [17], and a comparative study has shown that the design of the curriculum is associated with different levels of engagement with scientific methodology [18]. To our knowledge, this is the only survey across all German medical faculties regarding the medical students’ attitude towards scientific research. Consistent with a Croatian study, the students’ attitude towards scientific practice is positive overall [19]. International studies also indicate results consistent with our own, in that students think they are poorly prepared for scientific research [5], or misjudge their competencies [20]. Medical educators worldwide are establishing new teaching concepts and methodologies in order to motivate students for research [21–24]. As the gap between clinical practice and scientific research seems to be an international problem, the results of this study may be transferrable to other countries with a similar system of medical education.

The participants of this study would very much support the inclusion of scientific competency training, in the form of a small scientific project in their medical studies. These findings correspond to the international recommendations for medical education made by the World Federation for Medical Education (WFME) [1, 2] and the recommendations made by the German Council of Science and Humanities [25]. However, the medical students participating in our survey did not agree that scientific work would be an important part of their future clinical activities.

The majority of students participating in the survey do not consider scientific research as an important part of their future clinical practice and therefore, unsurprisingly, believe that a physician does not need to complete a doctorate. This is in contrast with the vast majority of participants who wanted to pursue a doctoral thesis (85.1%, *n* = 2380) (Table 1). Interestingly, the expectation that physicians should complete a doctorate is higher among students from earlier years of study, perhaps reflecting the public perception that physicians should hold the title *Dr. med.* in Germany.

**Table 2:** Most commonly mentioned suggestions for improvements of scientific training in medical curricula

Students should have more freedom of choice	“Personally, I am less interested in experimental scientific practice, nevertheless courses should be offered to interested students as an optional choice with an adequate level of intensity.”
Improvement of practical aspects of the training	“Please don’t overload the medical studies further with scientific work. Patient care should be prioritized over scientific research.” “Physician and researcher are different occupations! Rather provide physicians with soft skills and interacting with people and give researchers their own studies.”
Limit the medical curricula to the “essential”	“If scientific research is introduced into medical studies, the curriculum would have to be reduced. […] I think that many students would appreciate working on scientific research they are interested in, rather than agonize over enormous amounts of study matter.”
Critical interpretation of publications should be emphasized	“Comprehension and accurate interpretation of scientific research should be more important than conducting research independently.” “Interpreting study data (recognize good/bad quality), know how to access data bases for evidence based medicine for the clinical practice.”
Better supervision of scientific projects is required	“Good support is essential. […] Sadly, you are often left alone with your problems and get exploited as a student in scientific work instead of being encouraged.”
More interactive teaching formats are required	“A medical student memorizes far too much without questioning it. The discourse of medical teachers and students and also between students could be promoted through more interactive methods of teaching.” “I would not like to have more teaching, but self-reliant learning and classes with focus on student-centered activities should be promoted. Our studies are based on too much frontal teaching with power point. Discussion and direct application would have a greater effect.”

Surprisingly, almost one of every six students (13.9%) claimed to be working on a doctoral thesis, but did not claim to have gained scientific experience. This gap suggests that a significant number of students do not feel that they gain sufficient scientific competencies during their doctorate. In contrast, other surveys conducted in Germany [26] have indicated that most students who have successfully submitted their thesis consider their work as beneficial and would recommend pursuing a doctorate to other students. Nevertheless, there are almost no published data on the fraction of students who have started but not completed their projects. Further research is needed to determine the amount of terminated projects and the reasons for this. This is seemingly being viewed rather diversely by students and supervisors [27].

The publication of our results falls in a time of lively discussion about the quality and future of the medical doctorate in Germany. Schools and institutions, such as German Council of Science and Humanities, consider different strategies to improve the quality of medical doctorates. One approach aims to restrict the work on the doctoral thesis to postgraduate students only [28]. Others include the introduction of a PhD-equivalent either as structured doctoral studies [29, 30] or a mandatory scientific project in the final year of medical studies [31]. These mandatory courses could automatically lead to an MD-title being awarded upon successful completion of the medical studies, as is currently practiced in Austria [32]. In addition, international initiatives such as the *Organization of PhD Education in Biomedicine and Health Sciences in the European System (ORPHEUS)* [33] and the *Broadening Experiences in Scientific Training (BEST)* program [34] are currently being implemented and could serve as an inspiration to others. Further studies are needed to evaluate whether these approaches can really improve the current situation and quality of medical doctorates and motivate the students to pursue research careers.

All medical curricula in Germany are legally required to entail basic science teaching, including practical training [35], and there was no desire for more training in this area expressed by the students. However, only close to half of the students wished for more training in statistics, even though about 70% of the students rated their statistical competency as insufficient. The responses regarding Journal Club sessions were similar. The majority of students considers these sessions as helpful in enhancing their scientific competencies, yet about 20% of the students recommended having even less of these sessions. Journal Club sessions are a rarity in most of the current medical curricula in Germany, so one reason for the students’ apprehension might be a perceived increase in mandatory classes and assignments, as was frequently expressed in individual free-text responses. This is consistent with previous findings that medical students already suffer from high levels of stress [36–38]. It seems to be essential that medical schools adjust the design and content of their curricula to provide space where students can spend enough time on research [22]. Otherwise, students may be overloaded and demotivated to pursue additional scientific research training.

### Limitations

The high number of students who participated in our survey were generally representative of the body of German medical students as a whole. Our observed gender distribution (40% males and 59% female participants (Table 2)) closely represents the general gender distribution among medical students in Germany (39% male, 61% female) [39]. However, the survey captured only 2.7% of all medical students in Germany (87,863 at the time of our study). This is due to the fact that we had no possibility to directly contact every German medical student. Additionally, not all local medical student committees forwarded our survey invitation to students via their mailing lists. As a result the number of participants varies between different schools (Additional file 2: Figure S1).

The use of an anonymous and voluntary online questionnaire involves several other limitations. Since the survey was voluntary, participants may have rather been those with a higher interest in this topic leading to more favorable answers regarding the importance of scientific competencies. Additionally, multiple participation cannot not be ruled out. In the introductory text of the questionnaire, we made clear that the survey only addressed German medical students and participants had to select their medical school from a dropdown menu. However, we cannot rule out that some survey participants may not have been German medical students.

The questionnaire itself also had some potential limitations. Scientific competencies were assessed by students’ self-evaluation, so to increase the validity of our results, we would have to look at a more objective form of evaluation. Furthermore, some students may have misunderstood the terms *scientific work* and *scientific competency training* as *laboratory work* and *training in basic sciences*. Assuming that many medical students are less interested in laboratory work and basic sciences, as this study suggests, this may have influenced students’ responses to be less favorable.

It has to be noted that our survey only addressed German medical students. Additionally, the questions regarding the doctoral degree *Dr. med.* Refer to a local problem in Germany. Thus, the findings of our study are specific to medical students in Germany. Because of differences between educational systems, the findings have limited applicability when drawing comparisons to medical students’ opinions in other countries. However, most of the survey items addressed the importance of scientific competencies in general (e.g. for work as a physician) and the question of how scientific competencies can be imparted during medical studies. These results may also be transferrable to other countries.

## Conclusion

Scientific competencies are of great importance to medical students in Germany. Students focus on competencies that they consider as most beneficial for clinical practice, such as the critical evaluation of research results, and therefore see conducting their own research as less important for their future practice. Despite the importance of scientific competencies, students do not feel the necessary provisions are currently met and recommended an increase in scientific competency training.

Our study shows that students are not lacking motivation for scientific work and have numerous ideas for enhancing scientific teaching opportunities. To achieve the best possible outcome in this respect, a holistic approach to scientific competency training seems essential. We suggest this holistic approach to be based on three pillars: (i) a scientific core curriculum, (ii) intracurricular research projects, and (iii) special research programs for students with a high level of interest in medical research. Future research is needed to evaluate whether this approach would lead to increased scientific understanding and subsequently improve patient care.

## Additional files

Additional file 1: This document is a pdf of the online questionnaire that we used in our study. The items were translated from German to English. (PDF 108 kb)

Additional file 2: **Figure S1.** The number of survey participants per medical school. This graph indicates the variability of numbers of participants between different medical schools. (TIFF 1004 kb)

## Abbreviations

BEST: Broadening Experiences in Scientific Training
DFG: Deutsche Forschungsgemeinschaft
MD: Medical Doctor
ORPHEUS: Organization of PhD Education in Biomedicine and Health Sciences in the European System
PhD: Doctor of Philosophy
WFME: World Federation for Medical Education
