# Supplementary material for: The importance of scientific competencies in German medical curricula - the student perspective
Source: BMC Med Educ. 2018 Jun 19;18:146. doi: 10.1186/s12909-018-1257-4 (PMC6006583; doi:10.1186/s12909-018-1257-4)
Supplement: Supplementary file 1 — This document is a pdf of the online questionnaire that we used in our study. The items were translated from German to English. (PDF 108 kb) [file 12909_2018_1257_MOESM1_ESM.pdf]

bvmd_scientific_competencies → bvmd 21.11.2017, 21:42

Seite 01

Welcome to our survey on the importance of scientific competencies in German medical curricula!

The impartation of scientific competencies during medical studies is subject to fundamental discussions. It has often been criticized that medical students do not gain sufficient scientific competencies for their future clinical or scientific work. Thus, many medical faculties attempt to establish programs to improve the impartation of scientific competencies during medical studies.

This survey aims to assess the medical students' attitude towards scientific competencies. We would be very pleased if you could fill out the following questionnaire. It will take about 10 minutes of your time.

Please note: Only fill out the questionnaire, if you are a medical student!

Best regards,

Tabea, Antonius and Simon
National Officers on Medical Education
German Medical Students' Association

Seite 02

1. Age

What is your age?

[ ]

2. Gender

- ○ male
- ○ female
- ○ not specified

3. University

At which of University do you study?

[Please choose]

4. Semester

What is your semester?

5. Do you have scientific experience (work on scientific projects, publications etc.)?

Multiple answers possible.

- ☐ Yes, within the regular medical curriculum.
- ☐ Yes, from preparing a doctoral thesis.
- ☐ Yes, within a student project.
- ☐ Yes, as a student assistant.
- ☐ None.
- ☐ Other

6. Did you start working on a doctoral thesis?

- ○ I plan to work on a doctoral thesis.
- ○ I have started to work on a doctoral thesis.
- ○ I cancelled the work on a doctoral thesis.
- ○ I don’t want to do a doctorate.
- ○ I don’t know yet.

Seite 03

7. I rate my own scientific competencies as...

	very good	rather good	rather bad	very bad	not specified
Literature search	○	○	○	○	○
Critical analysis of scientific publications	○	○	○	○	○
Statistics	○	○	○	○	○
Scientific writing	○	○	○	○	○

8. Please state your level of agreement.

	fully agree	rather agree	rather not agree	fully not agree	not specified
The critical analysis of scientific publications is a key competeny for physicians.	○	○	○	○	○
The ability of conducting research projects independently is an important competence for physicians.	○	○	○	○	○
It is important that physicians do scientific work besides patient care.	○	○	○	○	○
Every medical student should conduct a research project as part of his/her curriculum (e.g. term paper, small research project).	○	○	○	○	○
There should be special programs for students who are interested in research (e.g. MD/PhD programs, research tracks).	○	○	○	○	○
My studies prepare me well for working on my dissertation.	○	○	○	○	○
Physicians should obtain a doctorate.	○	○	○	○	○

Seite 04

9. Of the following curricular contents I wish for...

	much more	more	equal	less	much less	not specified
“Good scientific practice”	○	○	○	○	○	○
Use of scientific publications in English	○	○	○	○	○	○
Critical analysis of scientific publications	○	○	○	○	○	○
Literature search	○	○	○	○	○	○
Journal Clubs	○	○	○	○	○	○
Study design	○	○	○	○	○	○
Laboratory methods	○	○	○	○	○	○
Statistics	○	○	○	○	○	○
Scientific writing	○	○	○	○	○	○

10. What is the best time point to start scientific research training during medical studies?
(optional)

Seite 05

11. Which aspects are important and have to be considered when setting up scientific education?

(optional)

12. Do you have general suggestions to improve the impartation of scientific competencies during medical studies?

(optional)

13. Captcha

seven plus one is

Letzte Seite

Thank you for participation!

We would like to thank you for your help.

Your answers have been saved. You may close your browser window now.

Antonius Ratte, Universität Heidelberg – 2017
