# Supplementary figures and images for: The importance of scientific competencies in German medical curricula - the student perspective
Source: BMC Med Educ. 2018 Jun 19;18:146. doi: 10.1186/s12909-018-1257-4 (PMC6006583; doi:10.1186/s12909-018-1257-4)

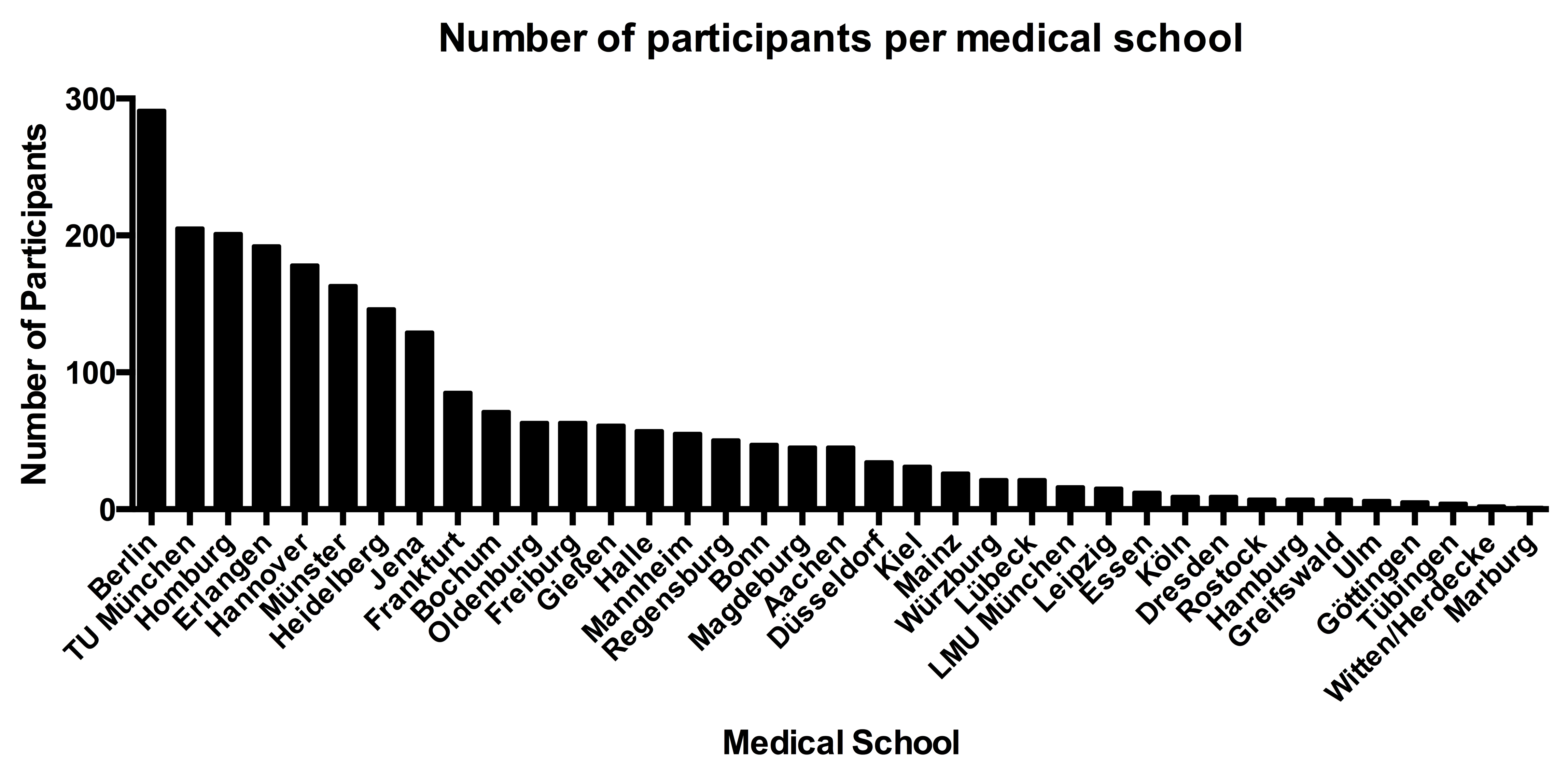

Supplement: Supplementary file 2 — Figure S1. The number of survey participants per medical school. This graph indicates the variability of numbers of participants between different medical schools. (TIFF 1004 kb) [file 12909_2018_1257_MOESM2_ESM.tiff]
